# Ameloplasty is counterproductive in reducing microleakage around Resin Modified Glass Ionomer and Resin based fissure sealants

**DOI:** 10.12669/pjms.36.3.1268

**Published:** 2020

**Authors:** Tabinda Nawaz Khan, Farhan Raza Khan, Syed Yawar Ali Abidi

**Affiliations:** 1 Dr. Tabinda Nawaz Khan, BDS, MDS. Assistant Professor, Department of Science of Dental Materials, Dow Dental College, Dow University of Health Sciences, Karachi, Pakistan; 2 Dr. Farhan Raza Khan, BDS, MS, MCPS, FCPS. Associate Professor, Operative Dentistry Dental Section, Department of Surgery, Aga Khan University, Karachi, Pakistan; 3 Dr. Syed Yawar Ali Abidi, BDS, FCPS. Professor & Head, Dept. of Operative Dentistry, Sindh Institute of Oral Health Sciences, Jinnah Sindh Medical University, Karachi, Pakistan

**Keywords:** Ameloplasty, Microleakage, Sealants, RMGIC, Flowable composite resin

## Abstract

**Objective:**

To compare the microleakage around resin modified glass ionomer cement (RMGIC) based sealants and flowable resin based sealants placed with or without ameloplasty in extracted human teeth.

**Methods:**

This in-vitro experimental study was conducted at the Operative Dentistry Department, Dow University of Health Sciences, Karachi, Pakistan from June 2017 to December 2018. Sixty extracted human molars and premolars were assigned to four groups (n=15) each, according to the type of fissure sealant (flowable resin based sealant or resin modified glass ionomer based sealant) used and either placed with or without ameloplasty. Specimens were thermocycled and then immersed in 1% methylene blue for 24 hours. Specimens were then sectioned and examined using stereo-microscope (50X) for microleakage that was scored on an ordinal scale. Mann-Whitney U test and Ordinal regression were applied. Level of significance kept at 0.05.

**Results:**

There was a statistically significant difference (p-value <0.001) between the two sealant types for the microleakage scores. Sealants placed with ameloplasty demonstrated significantly higher microleakage values (p-value <0.001).

**Conclusion:**

Microleakage was found to be more pronounced in RMGIC based sealants compared to the resin based sealants. Ameloplasty resulted in higher leakage around the sealants irrespective of the chemistry of material.

## INTRODUCTON

Occlusal surfaces of posterior teeth are considered to be a common site for the development of dental caries.^1^ The geometry of the fissure pattern at the occlusal surface favors the plaque accumulation, microbial growth and acidogenic activity necessary for carious attack.^1^

A number of preventive measures can be taken to limit the progress of dental caries.^2^ These include dietary modification, improved oral hygiene care, frequent brushing, flossing and use of fluoride containing dentifrices.^1^,^2^ Topical applications of concentrated fluoride gel or varnish have their role in managing dental caries in high-risk subjects.^3^ In addition to these, use of pits and fissures can be a valuable service, if done properly.^4^,^5^ Fissure sealants offer resistance against dental caries by physically occluding the deep and narrow fissures and imperfections on the tooth surface thereby denying the bacterial colonization to occupy the susceptible ecologic niches that are otherwise needed to initiate the cariogenic process.^4^,^5^

An important factor in enhancing the effectiveness of fissure sealants is the degree of its adaptability in the tooth substance.^6^ Apart from the choice of correct fissure sealant, a number of measures have been suggested to improve sealants adaptability, including ameloplasty.^6^ Ameloplasty is a technique that involves mechanically modifying the fissures anatomy by opening up the superficial part of enamel by using high speed burs thereby improving the penetrability of the sealant material into the fissure space.^6^

Out of various types of fissure sealant materials, two types are popular in the dental practice. These are composite resin based sealants and glass ionomer based sealants.^7^ Of these, resin based sealants are widely used.^7^ Ameloplasty (also known as enameloplasty or tooth fissurotomy) has been recommended to improve the retention of sealants. However, it’s not known whether ameloplasty has an additional benefit in reducing the microleakage around fissure sealants. We hypothesized that ameloplasty improves the sealing ability of fissure sealants. The objective of the present study was to compare the microleakage around resin modified glass ionomer (RMGIC) based sealant with that of flowable resin based sealant (RBC) placed with or without ameloplasty.

## METHODS

An in vitro experimental study was conducted from June to December 2018 at Operative Dentistry department, Dow University of Health Sciences, Karachi, Pakistan. The study protocol was approved by the ethics review committee of the Dow University of Health Sciences, Karachi, Pakistan. The reference # is IRB-252/DUHS-11.

Sixty extracted human molars and premolars (that were extracted due to orthodontic or periodontal reasons) were randomly assigned into two groups, RMGIC or RBC sealants. Written informed consent was taken at the time of extraction from the patient implying the donation of extracted teeth for research purposes. Collected teeth were further divided into two sub-groups of ameloplasty versus no ameloplasty, resulting in four groups of n=15 teeth.

Teeth with malformation, cracked or fractured crown, any pathological lesion, caries, erosion, restoration or attrition were excluded from the study. Collected teeth were kept in normal saline at 4°C for storage purpose after cleaning them with pumice water.

Ameloplasty was done by one of the investigators (TNK) using small pear-shaped diamond bur no.330 (Swiss Tec, Switzerland) running in a high-speed hand piece keeping the bur perpendicular to the long axis of the tooth. In this manner, the diameter of the bur (0.80mm) dictated the dimensions of ameloplasty, i.e. taper of 8 degrees and depth of 1mm was produced.

Sealant materials included a light-cured RMGIC (Vitremer; 3M-ESPE, St. Paul, MN, USA), and a flowable resin based sealant (Filtek Flow; 3M-ESPE, St. Paul, MN, USA). The sealant materials were applied according to the manufacturer’s direction. For both groups, teeth were treated with 37% phosphoric acid etchant. For RBC; the sealant material was air thin and light cured for 20 seconds after applying the adhesive (Adper Single Bond; 3M-ESPE, St.Paul, MN, USA). For RMGIC, the powder and liquid were mixed in ratio of 1:2 and was carried in a compule tip gun (635105, DENTSPLY, USA) and placed on the tooth fissure followed by light cured for 40 seconds.

Two controlled digital water bath (Human Lab Instrument Co, Korea) along the crushed ice container maintaining the temperatures of 600C ± 20C, 370C ± 20C and 40C ± 20C, with dwell time of 30 seconds were used for thermo-cycling purpose.For evaluating microleakage, dye penetration technique by 1% methylene blue was used. Specimen teeth were immersed for 24 hours at 37°c Before immersing into dye, the teeth were sealed apically and also two coats of nail paint were used on all surfaces except occlusal.

After washing and drying, the teeth were embedded into epoxy resin and section bucco-lingually in such a way that we obtained four slides for inspection from each tooth thereby total 222 slides were examined from 60 teeth (15 in each group) as shown in Table-I. The microscopic readout (50X) was done at the NED university of Science & Technology, Karachi, Pakistan.

**Table-I:** Distribution of study specimens (n=222) with their respective microleakage scores.

Sealant material	Intervention	No. of Teeth	Premolar: Molar	No. of Slides	Microleakage Scores				Mean rank	SD

					0	1	2	3		
RMGIC	Ameloplasty	15	8:7	60	0	6	9	45	167.28	4.85
	No ameloplasty	15	7:8	57	0	3	27	27	145.36	4.98
RBC	Ameloplasty	15	9:6	50	32	7	5	6	67.10	5.31
	No ameloplasty	15	8:7	55	39	7	8	1	55.90	5.06
Total		60	32:28	222	71	23	49	79		

Out of 240 slides, n=18 slides were excluded due to processing error.

An ordinal rating scale was used for evaluating microleakage.^8^ A single trained dentist assessed the microleakage scores using the following scale:

Score 0 = Good: No dye penetration visible,

Score 1 = Fair: Dye penetration up to the half of the fissures,

Score 2 = Poor: Dye penetration more than half of the fissure, not including the dentine,

Score 3 = Very Poor: Complete penetration into the underlying fissures.

For computing sample size, we used a previous study as a reference.^9^ The mean score of glass ionomer based sealant was 1.27±1.01 and for resin based sealant it was 0.82±1.19. Keeping this difference at confidence level 0.95 and power of test of 0.8, the sample size requirements turned out to be 55 teeth. Sample was inflated to 60.

For data analysis, Mann-Whitney U test was applied for the comparison of microleakage around two sealant materials. The effect of ameloplasty on microleakage was assessed using ordinal regression equation. Level of significance was kept at 0.05.

## RESULTS

The descriptive statistics of microleakage scores are shown in Table-I. The greatest microleakage was observed around RMGIC based sealants placed with ameloplasty (mean ranks: 167.28±4.85) and the least was observed for resin based sealants done without any ameloplasty (mean ranks: 55.90±50.6). There were 32 premolars and 28 molars assigned to the treatment groups. (Table-I)

A highly significant difference was observed for microleakage score in the two sealants type. RBC sealants were found to be better than RMGIC sealants. Similarly, there was a highly significant difference (*p*-value 0.001) in the microleakage scores for sealants placed with or without ameloplasty (irrespective of the material chemistry). Ameloplasty exerted a negative influence as sealants placed with ameloplasty exhibited poor microleakage. (Table-II).Regression equation suggests that sealants placed without ameloplasty yield the least microleakage scores (Table-III).

**Table-II:** Comparison of microleakage scores around RMGIC based sealants and flowable composite based sealants placed with or without ameloplasty.

Intervention	Sealant	Mean rank	95% Confidence Interval		p-value

			Lower Bound	Upper Bound	
Ameloplasty	RMGIC (n=60)	167.28	157.71	176.84	<0.001
	RBC (n=50)	67.10	56.62	77.57	
Without Ameloplasty	RMGIC (n=57)	145.36	135.55	155.18	<0.001
	RBC (n=55)	55.90	45.91	65.90	

n= number of slides, Mann-Whitney U test was applied,

RMGIC: Resin modified glass ionomer based sealants, RBC: Resin based composite sealants

**Table-III:** Effect of sealant chemistry and ameloplasty procedure on the microleakage scores.

Variables	Estimate	SE	Wald	p-value
Microleakage = 0	-3.86	0.40	92.82	<0.01
Microleakage = 1	-2.92	0.35	68.24	<0.01
Microleakage = 2	-1.00	0.25	15.89	<0.01
Sealants	-4.21	0.39	111.89	<0.01
Ameloplasty	-0.94	0.28	10.92	0.001
No Ameloplasty *	0			

* Reference category, SE: Standard Error, Ordinal regression was applied.

## DISCUSSION

The sealing ability of the restorative materials is the most important factor against the microleakage.^6^ Literature suggests that RBC sealant has an excellent adaptation to the tooth substance.^7^ in the present study, RMGIC based sealants were compared with RBC sealants. The latter is considered as the gold standard for fissure sealants.

Our results show that RMGIC exhibited greater microleakage scores as depicted by high degree of dye penetration; mean rank: 167.28±4.85 (as shown in Table-II). This can be attributed to the desiccated nature of the glass ionomer material that resulted in cracks throughout the occlusal surface of the sealant. This has also been observed in other studies as well^10^.^11^ and probably serves as the most appropriate explanation of the results in the present study. It’s known that RMGIC is a brittle material especially in thin cross-sections. Thus, it’s not unlikely that such thin layer of RMGIC get fractured under mechanical stress.^10^

Many attempts have been made to improve the adhesion of sealant restoration in the tooth and to make restoration leakage-free. But no material or technique has demonstrated an absolute success in this regards.^12^ Ameloplasty has been advocated to improve the adhesion of sealant material to the tooth structure. However, its effectiveness has remained questionable.^13^-^16^ An argument given against ameloplasty is that it worsens the microleakage.^16^ This is consistent with the findings of the present study. Contrary to this, others favor ameloplasty suggesting that superior outcomes can be achieved with sealants, if done properly.^14^ However, some investigators have observed neither any harm nor any additional benefit of ameloplasty procedure.^13^ The present study showed that ameloplasty has an adverse effect on the sealant materials resistance against microleakage. Both RMGIC and RBC sealants demonstrated poor microleakage when placed along with ameloplasty. When the effect of both the sealants and ameloplasty were studied together, the regression equation suggested that carrying out “no ameloplasty” yields the least microleakage scores, indicating a protective effect of not manipulating the enamel for any sealant placement (Table-III).

There are several ways of carrying out ameloplasty; these include use of fissure burs, round burs, air abrasion or even lasers. In the present study, ameloplasty was done using small pear shaped bur. However, no beneficial effect was observed in the present study for ameloplasty irrespective of the sealant material. In fact, it resulted in higher microleakage around sealant material. Why ameloplasty did not proved to be beneficial despite of increasing the surface area for adhesion, is a question of interest. A probable explanation would be that ameloplasty invariable increased the C-factor and hence increased the polymerization stresses in the sealant material.^17^ This would have resulted in deterioration of the bond at tooth-sealant interface and thereby increase in the microleakage.

Microleakage adversely affects the retention of the sealants. There are a number of methods used for determining microleakage. These tests include use of color producing radioactive isotopes, neutron activation analysis, air pressure method, electrochemical studies, scanning electron microscopy, chemical tracers, thermal and mechanical cycling and dye penetration studies.^18^ However, a universally accepted method for assessment of leakage is yet to be established. We employed dye penetration technique for the ascertainment of microleakage. It is one of the simplest, time-tested and economical methods for studying microleakage.^19^,^20^ In this respect, 1% methylene blue dye^20^,^21^ was used in the study. This dye was used as indicator as it’s not only easily assessable under visible light but being water-based, soluble and above all, it’s quality of not absorbed in or adhered to dentine matrix, the chances of false positives readouts are low.^22^ Microleakage was analyzed using linear leakage method. The dye molecules were smaller than the microbes, therefore, the likelihood of having false positive results were still there.^23^

In this study, microleakage was evaluated on an ordinal scale. The quantitative methods to assess microleakage are also available but these are more expensive, time consuming and difficult to employ therefore, qualitative method was chosen.^24^ Nonetheless, qualitative approach is well documented and accepted method of evaluating microleakage.^24^

**Fig.1:**
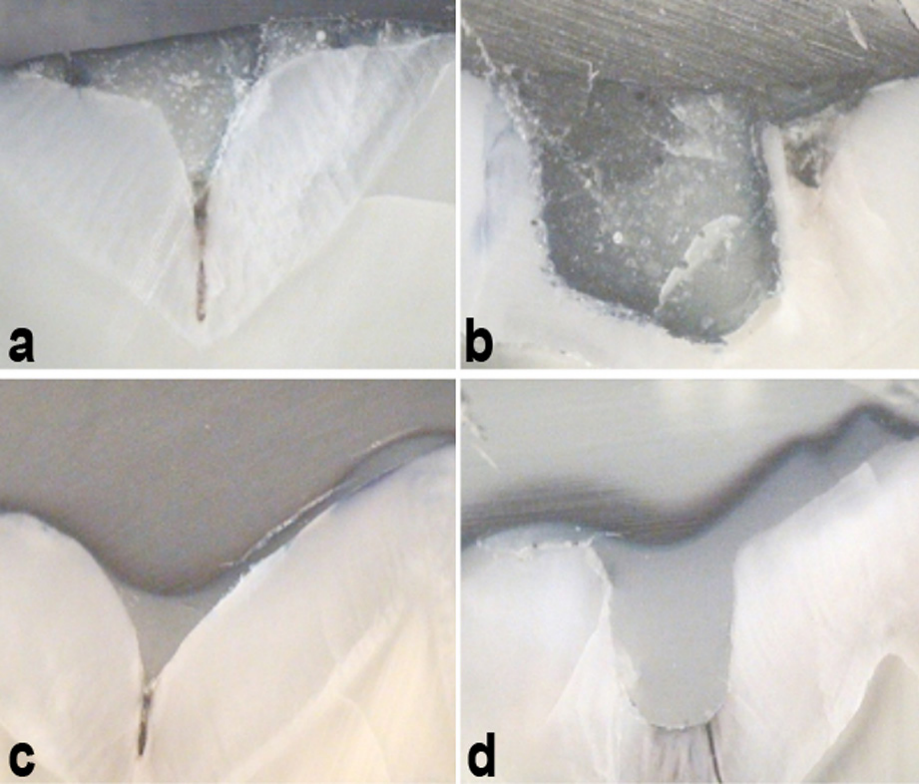
Section of teeth at 50X magnification exhibiting microleakage around sealant material. a: Resin modified glass ionomer sealant without ameloplasty, showing Grade 1 microleakage. b: Resin modified glass ionomer sealant with ameloplasty, showing Grade 3 leakage. c: Resin based sealant without ameloplasty, showing Grade 0 microleakage. d: Resin based sealant with ameloplasty, showing Grade 0 microleakage.

Compared to RMGIC based sealants, resin based sealants exhibited low microleakage scores. This could be attributed to the fact that flowable resins have a low contact angle at substrate^25^ and hence adapt well to enamel at the walls of fissures. Fig-1 Moreover, compared to RMGIC, the flowable resin forms a stronger micromechanical bond with the tooth structure.^11^

The clinical relevance of the present study is that ameloplasty has been advocated to increase the surface area for sealant adhesion and retention but our data suggests that it has no beneficial value. We hypothesized that ameloplasty improves the sealing ability of fissure sealants but our results show that ameloplasty resulted in significantly higher microleakage scores around RMGIC based sealants as well as the resin based sealants. Thus, it can be inferred that ameloplasty is counter-productive in improving the resistance of sealant material against microleakage.

To determine the reliability of microleakage scores, all the slides were re-examined by the second examiner (SYAA). Kappa statistics showed a good inter-examiner agreement (k= 0.82). Regarding limitations of the study, it’s important to note that it was an invitro experiment where ameloplasty was done using one type of bur only. Only two sealant materials were compared in this study. Despite of employing thermocycling, no efforts were done to simulate the occlusal/masticatory forces on the study specimens. The anatomy of the tooth (premolar versus molar) could act as a confounder on the sealant placement and hence could be affected by microleakage. The molars and premolars were randomly allocated to the treatment groups. This randomization took care of the confounding effect thus the tooth morphology did not influenced the microleakage. Lastly, the findings of such in vitro studies have to be endorsed using properly designed randomized controlled trials.

## CONCLUSIONS

Microleakage was significantly higher around RMGIC based sealants compared to the resin based sealants. Specimens treated with Ameloplasty showed higher microleakage compared to specimens with no ameloplasty suggesting that ameloplasty procedure does not improve the sealing ability of the fissure sealant material.

### Authors’ Contribution:

**TNK:** Conceived the Idea, carried out drafting and data collection. Furthermore, as the principal author, responsible and accountable for the accuracy and integrity of the work.

**FRK:** Took the responsibility of data analysis and critical review.

**SYAA:** Did Supervision of the project and manuscript review.
